# Exploring new vistas in biomedical journal publishing

**DOI:** 10.2349/biij.2.4.e51

**Published:** 2006-10-01

**Authors:** BJJ Abdullah, KH Ng

**Affiliations:** Department of Biomedical Imaging (Radiology), Faculty of Medicine, University of Malaya, Kuala Lumpur, Malaysia

To write what is worth publishing, to find honest people to publish it, and get sensible people to read it, are the three great difficulties in being an author (Charles Caleb Colton, English sportsman and writer, 1780-1832).

Scholarly journals have been the basic tool for scientific communication for over three centuries, since the Flemish itinerant craftsman William Caxton and his assistant Wynkyn de Worde set up his heavy wood and metal German designed printing press in the shadow of St Paul’s Cathedral in 1476. Scientific societies publish and distribute these journals to their members as a part of their subscription. With the huge investments in basic scientific research which occurred after the Second World War, the scientific societies had great difficulty in keeping pace with the phenomenal growth in the number of publications that sprouted. Journals catering to new disciplines arose when researchers in these specialised areas found it difficult to get their works published in the journals which kept to traditional views of the boundaries of disciplines [1]. Publishers, in general, ceased to be their own booksellers in the 18^th^ century and their own printers in the 19^th^ century. In the 20^th^ century, they coped with a remarkable technological shift in printing and book manufacturing technology from hot metal to cold setting.

In the 21^st^ century, the focus in publishing is on information management and not technology. Publishers create content, and they manage, add value to, stretch, recycle, translate, and transmit it. In addition, they have been successful from a very early stage in distancing themselves from delivery technology and leaving the task of managing ever-obsolescing technology, the mechanics of distribution and delivery, to others.

Despite these achievements, publishers and journals face challenges on account of the following:

## GLOBALISATION

Research is increasingly becoming more international and more collaborative. This has been driven by the scientific advantages of sharing knowledge and know-how beyond a single institution; lower costs of air travel and communication; increased use of information technology; national policies encouraging international collaboration; need for higher international standing,; and graduate student study abroad programmes. This trend is reflected in both an increase in the average number of authors and institutions on an article, and in the proportion of foreign addresses. [2]. The average number of co-authors per article published in EU increased from 3.33 to 4.81 between 1988 and 2003, while articles with at least one co-author from a non-EU country accounted for 36% of all articles in 2003, up from 17% in 1988 [3].

## INTELLECTUAL PROPERTY RIGHTS

From the perspective of the publishing industry, the basis of all content management is a clear system of ownership and reward for use. Despite the Web, the industry feels that information never has been, never could be, and never should be free. They also contend that the value of information depends both on its intrinsic nature and its timeliness. It has to be both authoritative (accurate, true) and accessible. What publishers do essentially is guarantee the former and ensure the latter.

### Technological change

Publishers have always dealt with this quite effectively and many new technologies favour publishers. ‘Watermarking’ technologies for identifying text-ownership, for example, the Digital Object Identifier enable flexible, trackable dissemination of published works in many forms and media. They will certainly widen access and use. Print-on-demand digital printing that allows print runs as low as five being economical means that no book need ever go out-of-print again. Use of this technology will make it possible for any book to be individually custom-printed and bound for a customer at a bookshop terminal while they wait.

### The Internet and the World Wide Web

They enable almost instant access to millions of pages of information, entertainment, and education. This has added to the challenges of scientific publishing [4]. The major attractions of the electronic publishing of scholarly monographs are easier access and visual processing or an enhanced intellectual content. The electronic linking to additional resources or the provision of moving images, for example, a rotating anatomical image, clearly are beyond the capabilities of the printed book and are definitely added value [5].

However, some argue that [6] publishing is not just about being able to access information. The reader’s engagement with the printed page is a much more subtle and active relationship. Some would go so far to equate it to an aesthetic, almost mystical experience, in which the author and the reader commune. The reader brings almost as much to the experience as the author, which is unique to the medium. Therefore, the expressions like “lost in a book”.

In addition, issues of authenticity and confidence which are particularly important to users, is another downside cited for electronic publishing. Without a physical artefact, how sure are the readers that what they are viewing is the original document as intended by its author, owner or publisher without a physical artefact? Graham has identified the possibility of three types of document change [7]:

- Accidental – loss of the final version and changes made during copying;
- Well-intentioned – updates, restructuring;
- Fraudulent – changes of one’s own work to cover one’s tracks or change of evidence for a variety of reasons, or damage to the work of another.

Despite the gloom and the doom of electronic publishing and the Internet, publishing in general has shown that it is a dynamic, growing industry. It is estimated that worldwide annual revenues generated from English-language Scientific, Technical and Medical journal are estimated at around USD 5 billion in 2004 [2]. The industry employed over 90,000 people of which about 40% or 36,000 are employed in the EU. In addition, an estimated 20-30,000 full time employees are indirectly supported by the STM industry globally (suppliers, freelancers, external editors, etc) [2].

As researchers and biomedical professionals, we all agree on the importance of research in our society. However, we are also aware that the values accruing from the research process are dependent on others being able to use this information and even further its benefits. Therefore, publishing has an important role in being a medium for the dissemination of knowledge. However, as with any medium of communication, there is an urgent need for raising the level of awareness and skills of editors, reviewers, and authors in the developing countries.

To re-examine publishing in the biomedical arena, a Workshop on Publishing for Biomedical Journal Editors and Reviewers: Publishing in a global competitive world (3rd International University of Malaya Research Imaging Symposium) was held in Kuala Lumpur, Malaysia. The workshop was organised by the University of Malaya Research Imaging Centre (UMRIC) and Biomedical Imaging and Intervention Journal (***biij***). The major stakeholders in the biomedical publishing world, authors, editors, reviewers, copyeditors, designers, publishers, and librarians participated in this very important workshop. It set benchmarks in biomedical publishing. There were more than 100 delegates from 14 countries.

The objectives of this workshop were:

- To improve the skills of editors and reviewers
- To review the current status of biomedical journal publishing
- To assess the problems and constraints facing biomedical journals
- To develop guidelines for quality of biomedical journal publishing
- To promote a code of ethics for biomedical journal publishing
- To analyse trends in journal publishing
- To promote collaboration and networking among editors of biomedical journals

The meeting provided a forum for discussion and exchange of ideas and new developments. The need for biomedical journals to be current and relevant in the wake of rapid technological advancements and the question of how instantaneously should crucial clinical information be communicated to physicians and, ultimately, to their patients were raised at the workshop. How much publishing time should we, as medical editors, allocate for a medical research article before it is published? How long should the process of cataloguing, review, page layout, and design hold back crucial clinical results? Do we have definite answers to these crucial questions? Unfortunately, we do not. We also cannot be certain that we can provide the answers to these questions. There are just too many conflicting variables to take into account. However, we can be certain of the philosophy behind this complicated and lengthy process. That is, to ensure the publication of a world-class biomedical journal, keeping our professional integrity intact.

This common quest for professional integrity led to this opportune gathering of the major stakeholders in the biomedical publishing world. We have tasked some of the best talents in the field of biomedical publishing to discuss and create a comprehensive program that covers the entire process of publishing biomedical journals in today’s world.

The topics ranged from responsibilities of authors, editors and reviewers; the role of editors and reviewers; editorial independence and governance; the peer review process; publication ethics; manuscripts for the evidence-based medicine era; developments in international journal publishing; the art of copy editing; the publishing cycle to Open Access Online Publishing. All the presentations are available as AVI or MP4 files at http://www.biij.org/biomedical-imaging-intervention-journal-resources.asp

The co-sponsors of the meeting included British Medical Journal, Canadian Medical Association Journal, The College of Pathologists, Academy of Medicine of Malaysia, College of Radiology, Academy of Medicine of Malaysia, Elsevier, Hong Kong Medical Journal, Medical Journal of Australia, Medical Journal of Malaysia, Singapore Medical Journal, Taylor and Francis, and Thomson Scientific.

We are indeed witnessing a very exciting phase in biomedical journal publishing.
